# Large synteny blocks revealed between *Caenorhabditis elegans *and *Caenorhabditis briggsae *genomes using OrthoCluster

**DOI:** 10.1186/1471-2164-11-516

**Published:** 2010-09-24

**Authors:** Ismael A Vergara, Nansheng Chen

**Affiliations:** 1 Department of Molecular Biology and Biochemistry, Simon Fraser University, 8888 University Drive, Burnaby, B.C., V5A 1S6, Canada

## Abstract

**Background:**

Accurate identification of synteny blocks is an important step in comparative genomics towards the understanding of genome architecture and expression. Most computer programs developed in the last decade for identifying synteny blocks have limitations. To address these limitations, we recently developed a robust program called OrthoCluster, and an online database OrthoClusterDB. In this work, we have demonstrated the application of OrthoCluster in identifying synteny blocks between the genomes of *Caenorhabditis elegans *and *Caenorhabditis briggsae*, two closely related hermaphrodite nematodes.

**Results:**

Initial identification and analysis of synteny blocks using OrthoCluster enabled us to systematically improve the genome annotation of *C. elegans *and *C. briggsae*, identifying 52 potential novel genes in *C. elegans*, 582 in *C. briggsae*, and 949 novel orthologous relationships between these two species. Using the improved annotation, we have detected 3,058 perfect synteny blocks that contain no mismatches between *C. elegans *and *C. briggsae*. Among these synteny blocks, the majority are mapped to homologous chromosomes, as previously reported. The largest perfect synteny block contains 42 genes, which spans 201.2 kb in Chromosome V of *C. elegans*. On average, perfect synteny blocks span 18.8 kb in length. When some mismatches (interruptions) are allowed, synteny blocks ("imperfect synteny blocks") that are much larger in size are identified. We have shown that the majority (80%) of the *C. elegans *and *C. briggsae *genomes are covered by imperfect synteny blocks. The largest imperfect synteny block spans 6.14 Mb in Chromosome X of *C. elegans *and there are 11 synteny blocks that are larger than 1 Mb in size. On average, imperfect synteny blocks span 63.6 kb in length, larger than previously reported.

**Conclusions:**

We have demonstrated that OrthoCluster can be used to accurately identify synteny blocks and have found that synteny blocks between *C. elegans *and *C. briggsae *are almost three-folds larger than previously identified.

## Background

The conservation of large scale genomic sequences across two or more genomes --synteny blocks-- is of primary interest because their identification sets up a stage for identifying and characterizing sequence and functional differences among genomes [1]. The term synteny has been used in different contexts in the past. Originally, synteny was used to indicate the colocalization of different genes in corresponding chromosomes of different species (a.k.a. "chromosomal synteny") [2]. Recently, with the availability of thousands of sequenced genomes, synteny has been used to describe the conservation of co-localized genes in the same order within different genomes (a.k.a "conserved segment"). In some occasions, the term "conserved synteny" has been used to refer a genomic region in which the chromosomal location of multiple markers is conserved, but not necessarily their precise order [3]. The term "synteny block" [4] has been defined previously as a segment in one genome that can be converted, through genome rearrangements, into a conserved segment in another genome. As such, a synteny block does not necessarily represent areas of perfectly continuous similarity between genomes. In this paper, we use the term "perfect synteny block" as "a genomic region of perfectly conserved gene content, order and strandedness", as defined by Coghlan and Wolfe [5]. As an extension to this definition, we use "imperfect synteny block" as "a genomic region containing some level of interruption, and in which order and strandedness is not necessarily conserved" [6].

In the past decade, different methods have been proposed to identify synteny blocks [7-12]. However, these methods usually lack one or more of the following functionalities required for detailed analysis: (1) Comparing more than two genomes, (2) Allowing interruptions within synteny blocks; (3) Capturing the strandedness of genes; and (4) Addressing one-to-many orthologous relationships. Failure to provide these functionalities makes these programs inapplicable for the identification of genome rearrangement events such as inversions, insertions, reciprocal translocations and segmental duplications. To tackle these problems, we have recently developed a new method called OrthoCluster, a computer program for the systematic detection of synteny blocks between two or among multiple genomes [6]. Briefly, OrthoCluster takes as input genetic markers (such as genes and microsatellites) and their relationships (such as orthologous relationships) and scans through two or more genomes for synteny blocks. OrthoCluster distinguishes genetic markers as either in-map or out-map. A genetic marker in one genome is called in-map if it has orthologous genetic markers in all corresponding genomes. In contrast, a genetic marker in one genome is called out-map if it does not have orthologous genetic markers in corresponding genomes.

To facilitate the application of OrthoCluster, we have recently developed a web server called OrthoClusterDB [13]. Additionally, a book chapter describing its usage and application has been published [14]. In addition to its use in identifying synteny blocks, OrthoCluster can be applied to identify segmental duplications within a genome [15].

*C. elegans *is a free living soil-dwelling hermaphrodite nematode and a popular model organism for biomedical studies because of its small size, transparent body, short life cycle, ease of propagation and compact genome. *C. elegans *was also the first multicellular organism subject to whole genome sequencing [16], and the genome sequence of this species has been declared to be complete, with no remaining gaps in 2002. After more than a decade of annotation after its first publication, the genome of *C. elegans *is arguably the best annotated of a multicellular organism to date [17,18]. The sequencing of its sister species *Caenorhabditis briggsae*, also a hermaphrodite, sets up an excellent platform for comparative genomic analysis [5,19]. Recently, by applying OrthoCluster, we have identified segmental duplications in the nematode *Caenorhabditis elegans *genome, including a large duplication that is polymorphic among *C. elegans *laboratory N2 strains [15]. In this project, we applied OrthoCluster to identify synteny blocks between *C. elegans *and its sister species *Caenorhabditis briggsae*, whose genome was sequenced a few years ago [19].

Synteny block identification and characterization is critical for understanding genome structure and functional domains of genomes. Synteny between *C. elegans *and *C. briggsae *was first explored when the first sequenced reads of *C. briggsae *became available. Using their program WABA (for "Wobble Aware Bulk Alignment") [20], Kent and colleagues compared the whole genome sequence of *C. elegans *and 8 Mb of *C. briggsae *sequences (in 229 cosmids) and found that 59% of these genomes are homologous at the base level, while 41% of the genome sequences are found in nonalignable regions. Using these alignments, they estimated the synteny relationship between *C. elegans *and *C. briggsae *and found that ~40% of the genome is resistant to rearrangements. Later, using a gene-based approach, Coghlan and colleagues examined the slightly larger set of sequences (12.9 Mb of *C. briggsae *genome) for synteny blocks and genome rearrangement events [5] and found many perfect synteny blocks. They also identified larger imperfect synteny blocks between these two genomes with an average size of 53 kb. The completion of the *C. briggsae *genome sequencing project enabled the *C. briggsae *genome analysis group to compare *C. elegans *and *C. briggsae *at the whole genome scale at the supercontig level [19]. To identify regions of colinearity, the program WABA [20] was used to produce base level alignments, followed by merging of adjacent blocks and bridging of small transpositions and inversions. Eventually, 4,837 alignments were obtained that cover 84.6% of the *C. elegans *genome, with a median length of 5.6 kb (mean = 37.5 kb) [19]. The average size is smaller than that obtained using gene-based analysis reported previously [5]. Recently, a chromosomal-level assembly of the *C. briggsae *genome [21] has been constructed, which can be utilized to facilitate synteny identification and analysis. Here, taking advantage of this new assembly and our newly developed program OrthoCluster, we revisit and reanalyze synteny blocks between these two genomes.

## Results

### Initial comparison between *C. elegans *and *C. briggsae *genomes

**Figure 1:**
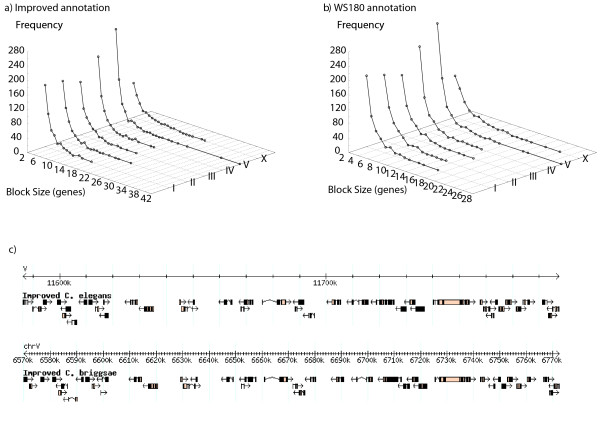
**Perfect synteny blocks in the *C. elegans *genome**. a) Size distribution for perfect synteny blocks obtained using the improved annotation. b) Size distribution for perfect synteny blocks obtained using the WS180 annotation. c) The largest perfect synteny block between *C. elegans *and *C. briggsae *obtained using the improved annotation.

**Figure 2:**
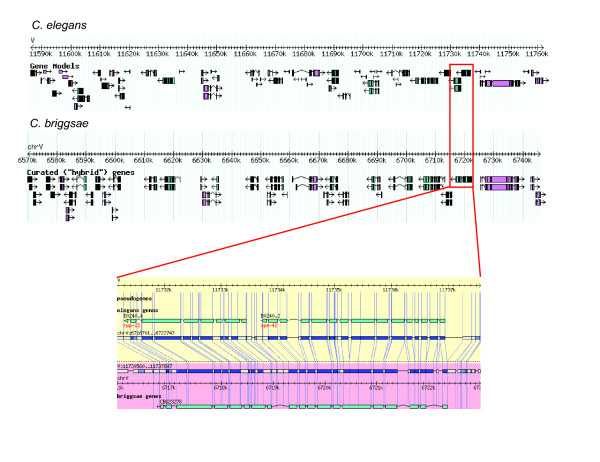
**An example of a defective gene model in *C. briggsae***. The alignment of the two adjacent genes B0240.4 and B0240.2 in *C. elegans *against CBG23278 in *C. briggsae*.

Using the *C. elegans *genome annotation in WormBase release WS180 [17], the genome assembly and annotation of *C. briggsae *[21] (from the same release), and the correspondence file generated using InParanoid [22], we detected 3,075 perfect synteny blocks between the genomes of *C. elegans *and *C. briggsae *using OrthoCluster. These blocks range in size from 2 to 28 genes (961 bp to 168.2 kb, Figure 1b). Examination of these synteny blocks, including the gene models contained within these blocks, immediately suggests that many gene models (primarily the *C. briggsae *ones) are defective, which leads to the unnecessary truncation of large synteny blocks. One example of such case is shown in Figure 2, which illustrates two genomic regions in *C. elegans *and *C. briggsae *that are nearly perfectly conserved with the exception of one gene in *C. elegans*, B0240.4, which breaks the synteny. Based on the current WormBase annotation (WS180), this gene does not have a clear ortholog in *C. briggsae*. Examination of the alignment of genes B0240.4 and B0240.2 in *C. elegans *and gene CBG23278 in *C. briggsae *(which is the predicted ortholog of B0240.2) suggests that the predicted *C. briggsae *gene is defective. Indeed, the current gene model of CBG23278 can be split into two separate genes, one orthologous to B0240.4 and the other orthologous to B0240.2. Experimental validation based on PCR reactions that prove the existence of the two genes and the non-existence of the junction on a cDNA library for *C. elegans *suggests that these are two separate genes (data not shown). Fixing cases like this will uncover many more *bona fide *orthologous relationships between *C. elegans *and *C. briggsae*.

### Synteny-based gene model correction and ortholog assignment

**Figure 3:**
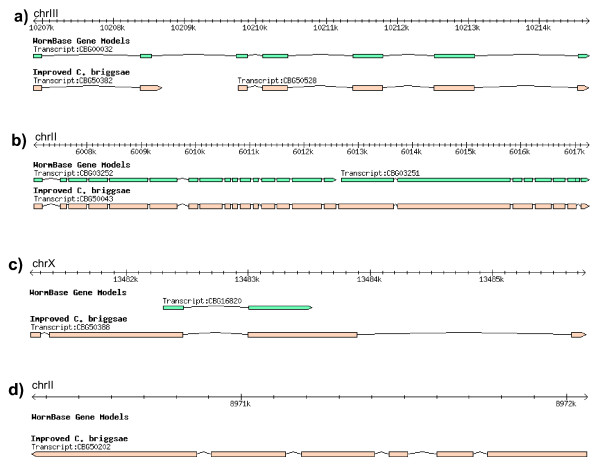
**Examples of revised gene models in *C. briggsae***. a) A gene model (CBG00032) is split in two gene models (CBG50382 and CBG50528). b) Two gene models (CBG03252 and CBG03251) were merged to form one new gene model (CBG50043). c) A gene model was replaced by a new gene model. d) A new gene model.

We developed a procedure (described in detail in Methods) in order to detect and correct defective gene models at the whole genome scale. Altogether, we identified 52 putative new genes in *C. elegans *(Table 1, Additional file 1). In contrast, in *C. briggsae*, we have generated 582 revised gene models, 191 of which correspond to novel gene structures in previously defined intronic or intergenic regions (Table 1, Additional file 2). Most deletions and additions were due to gene splits and gene merges (Figure 3). We assigned new orthologous relationships based on sequence similarity revealed by the improved gene annotation and synteny, which leads to the assignment of 949 new orthologous relationships (Table 2).

**Table 1:** Gene model improvement in *C. elegans *and *C. briggsae*

	*C. elegans*	*C. briggsae*
Initial number of genes	20,140	19,522

Outmap genes replaced by predictions	0	9

Split genes	0	130

Merged genes	0	250

Predictions added because of split genes	0	262

Predictions added because of merged genes	0	124

Genes added because of new genes	52	191

Genes deleted because of special cases	0	7

Predictions added because of special cases	0	5

Final number of genes	20,192	19,717

**Table 2:** Ortholog assignment between *C. elegans *and *C. briggsae*

	*C. elegans*				*C. briggsae*			
	**Improved annotation**		**WS180 Annotation**		**Improved annotation**		**WS180 annotation**	

	**Number**	**%**	**Number**	**%**	**Number**	**%**	**Number**	**%**

**Orthologous genes**	14,973	100	14,345	100	14,751	100	14,092	100

**Genes in one-to-one relations**	13,794	92.1	13,406	93.5	13,531	91.7	13,047	92.6

**Genes in one-to-many relations**	1,179	7.87	939	6.55	1,220	8.27	1,045	7.42

**Total orthologous relations**	17,818		16,869		17,818		16,869	

### Genome-wide identification and analysis of synteny blocks

#### Orthologous relationships

Based on the improved orthologous relationships (see Methods), the majority of the orthologous relationships between *C. elegans *and *C. briggsae *are one-to-one relationships (Table 3), with only 7.9% of the *C. elegans *genes with orthologous relationships (or 5.8% of the total genes in the improved annotation of *C. elegans*) having more than one ortholog in *C. briggsae*, ranging from 2 to 147 orthologs. Likewise, 8.3% of the *C. briggsae *genes with orthologous relationships (or 6.2% of the total genes in the improved annotation of *C. briggsae*) have more than one ortholog in *C. elegans*, ranging from 2 to 24 orthologs. One-to-one orthologous relationships exist mainly between homologous chromosomes of *C. elegans *and *C. briggsae *(Table 3), demonstrating strong chromosomal synteny, in good agreement with previous studies [21].

**Table 3:** One-to-one orthologous relationships between *C. elegans *(rows) and *C. briggsae *(columns).

Chromosomes	ChrI	ChrII	ChrIII	ChrIV	ChrV	ChrX	ChrUn	Total
**I**	**1519 (360)**	46 (2)	10 (2)	16 (0)	13 (0)	0	47	2,015

**II**	5 (3)	**1,698 (175)**	8 (0)	12 (0)	18 (1)	6	38	1,964

**III**	9 (1)	28 (0)	**1,741 (67)**	42 (0)	5 (0)	1	51	1,945

**IV**	27 (6)	11 (0)	34 (9)	**1,779 (51)**	21 (2)	20	67	2,027

**V**	9 (2)	10 (2)	14 (0)	17 (0)	**1,973 (249)**	6	74	2,356

**X**	8 (0)	7 (1)	2 (0)	2 (0)	4 (0)	**1,888**	11	1,923

**Total**	1,577 (372)	1,800 (180)	1,809 (78)	1,868 (252)	2,034 (252)	1,921	288	**12,230**

Numbers in parenthesis represent relationships found in the "_random" assembly of the chromosome, as reported by Hillier and colleagues [21].

#### Perfect synteny blocks

**Figure 4:**
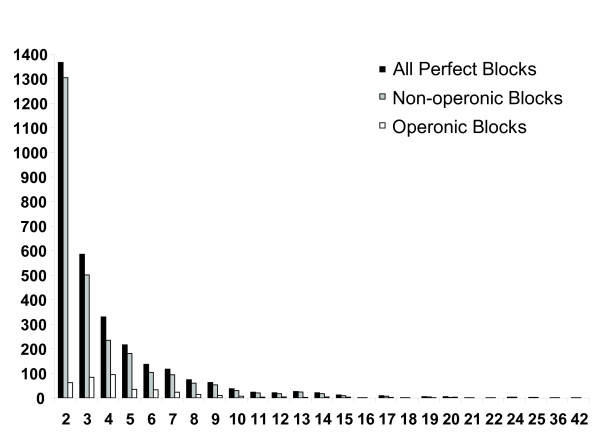
**Size distribution of perfect, non-operonic and operonic synteny blocks**. The size of a block is defined by the number of genes within that block.

Using OrthoCluster and the improved genome annotations, we identified 3,058 perfect synteny blocks (each synteny block contains at least two genes and no mismatches). Of these blocks, 2,687 are non-nested, whereas 371 are nested within larger synteny blocks. A nested synteny block corresponds to a subset of genes within a larger synteny block that is found duplicated in different genomic regions in either the same or different chromosomes. The largest perfect synteny block between the genomes of *C. elegans *and *C. briggsae *contains 42 genes (Figure 1a, Figure 4) and spans a 201.2 kb genomic segment in Chromosome V of *C. elegans*, corresponding to a 202.5 kb segment in Chromosome V of *C. briggsae *(Figure 1c). The mean size of these perfect synteny blocks span 18.8 kb, while the median size is 12.7 kb. Altogether, the perfect synteny blocks cover 11,058 genes in *C. elegans *(51.3 Mb, or 51.1% of the *C. elegans *genomic sequence) and 10,879 genes in *C. briggsae *(49.5 Mb, or 45.6% of the *C. briggsae *genomic sequence) (Table 4). Genome-wide view of synteny blocks can be generated using OrthoClusterDB [13] (Additional file 3, Figure S1). Most (2,770, or 90.6%) of the synteny blocks in *C. briggsae *are conserved within the homologous *C. elegans *chromosome thus showing strong chromosomal synteny (Table 5). Among the 288 synteny blocks (out of the 3,058 perfect blocks) in *C. elegans *that are mapped to a non-homologous chromosome in *C. briggsae*, 78.2% are located in Chromosomes II, IV and V of *C. elegans *and 72.9% are located in Chromosomes I, IV and V of *C. briggsae*.

**Table 4:** Perfect synteny blocks, operons, and their corresponding genomic coverage, size and range in *C. elegans*.

Chromosome	All perfect synteny blocks						Operons					
	
	Synteny blocks	Coverage (%)	Mean (Kb)	Median (Kb)	Range (Kb)	Range (genes)	Operons	Coverage (%)	Mean (Kb)	Median (Kb)	Range (Kb)	Range (genes)
**I**	445	53.4	18.3	13.6	[2.0-102.9]	[2-17]	246	16.5	10.2	8.0	[1.1-81.6]	[2-8]

**II**	525	47.1	16.0	11.3	[1.0-111.9]	[2-24]	203	11.2	8.4	6.3	[1.6-51.9]	[2-8]

**III**	442	54.1	18.1	14.5	[0.6-75.8]	[2-20]	264	17.5	9.1	7.6	[1.1-33.0]	[2-7]

**IV**	525	43.6	16.6	12.4	[1.0-95.7]	[2-20]	196	9.7	8.7	6.9	[1.1-46.7]	[2-7]

**V**	690	43.3	15.2	9.0	[0.9-201.2]	[2-42]	154	5.6	7.6	5.6	[1.6-41.5]	[2-7]

**X**	431	65.4	32.4	23.4	[1.0-168.2]	[2-25]	57	2.1	6.5	6.2	[1.7-23.6]	[2-3]

**Total**	3,058	51.1	18.8	12.7	[0.6-201.2]	[2-42]	1,120	9.8	8.8	6.8	[1.1-81.6]	[2-8]

**Table 5:** Distribution of perfect synteny blocks between *C. elegans *chromosomes (rows) and *C. briggsae *chromosomes (columns).

Chromosome	ChrI	ChrII	ChrIII	ChrIV	ChrV	ChrX	ChrUn	Total	Non-homologous
**I**	**325 (89)**	9 (0)	0 (0)	3 (0)	2 (0)	0	17	445	14

**II**	13 (0)	**370 (40)**	1 (2)	28 (1)	37 (2)	3	28	525	87

**III**	3 (0)	13 (0)	**364 (14)**	7 (0)	18 (0)	0	23	442	41

**IV**	13 (3)	8 (9)	2 (1)	**423 (12)**	22 (3)	10	19	525	71

**V**	17 (0)	3 (1)	1 (0)	41 (0)	**520 (72)**	4	31	690	67

**X**	0 (0)	2 (0)	0 (0)	0 (0)	6 (0)	**417**	6	431	8

**Total**	371 (92)	405 (50)	368 (17)	502 (13)	605 (77)	434	124	**3,058**	288

**Non-homologous**	46 (3)	35 (10)	4 (3)	79 (1)	85 (5)	17	N.A.	288	

Numbers in parenthesis correspond to the number of synteny blocks found in the "_random" assembly of the chromosome, as reported by Hillier and colleagues [21].

Perfect synteny blocks of different sizes are not evenly distributed in all chromosomes. Our results indicate that perfect synteny blocks on Chromosome X are significantly larger than those on the autosomal ones. The median length of perfect blocks within autosomal chromosomes is 11.8 kb (mean = 16.7 kb), whereas the median length of these type of blocks within Chromosome X is 23.4 kb (mean = 32.4 kb), more than two-folds larger (*p *<0.01, Mann-Whitney U test). This observation is consistent with previously reported observations [19,21], suggesting that Chromosome X is subject to fewer rearrangement events. Alternatively, most rearrangements occurring in Chromosome X are lethal and are therefore not preserved in evolution. Taking the definition of clusters and arms provided by Hillier and colleagues, we find that, within autosomes, the median length of perfect synteny blocks in autosomal centers is 11.6 kb (mean = 16.6 kb), whereas the median length of perfect synteny blocks in autosomal arms is 12.2 kb (mean = 16.9 kb). This difference is not statistically significant (p-value = 0.15, Mann-Whitney Test). Among all six chromosomes, the one with the highest genomic coverage is Chromosome X (65.4%). Chromosome V, which is the largest chromosome in *C. elegans*, also contains the largest number of blocks (22.6%).

Species-specific gene family expansions/contractions were observed previously and many gene family members have been found to form tandem clusters in *C. elegans *and *C. briggsae *[19,23], which is consistent to our recent observation that the *C. elegans *genome harbors a large number of intrachromosomal duplications, many of which occur in tandem [15]. In this project, we have demonstrated that members of a same gene family can form tandem clusters within synteny blocks identified using OrthoCluster. We found 534 such cases, in which 424 contain more genes in *C. elegans *while 110 have more genes in *C. briggsae *within these tandem gene clusters. One example of this is a syntenic region that has a higher presence of members of the GST (glutathione-S-transferase) family of genes in *C. elegans *than in *C. briggsae *(Additional file 4, Figure S2). Further exploration of these regions is required to unveil the mechanisms underlying the expansion/contraction of these genes.

Our gene model improvement has greatly enhanced our ability to identify larger synteny blocks. When we use the WS180 annotation (before gene model improvement) for the detection of perfect synteny blocks, we found more (3,075) but smaller blocks (Figure 1a, b; Additional file 5, Figure S3; Additional file 6) compared to those described above. For example, the largest synteny block contains 42 genes using the improved annotation, but only 28 genes if we use the WS180 annotation. In fact, the 28 genes are a subset of the synteny block composed of 42 genes detected using the improved annotation. Compared to the WS180 annotation, the improved annotations increase the coverage of the chromosomes (Additional file 6).

#### Contribution of operons to perfect synteny blocks

According to WormBase annotation (release WS180), there are 1,120 operons in *C. elegans*, ranging in size from two to eight genes (Table 4). Previous comparative studies have concluded that these operons are highly conserved between *C. elegans *and its sister species *C. briggsae*, with the vast majority of the operons (96% [19] and 93.2% [24]) conserved between these two species. What is the contribution of operons to the perfect synteny blocks identified between these two species? In order to address this question, we have examined the contribution of operons to perfectly conserved synteny blocks (Table 4, Figure 4). Our analysis suggests that operons constitute an insignificant part of the perfect synteny blocks.

First, the portion of the *C. elegans *genome covered by the 1,120 annotated operons (9.8%) is dramatically smaller than that covered by the 3,058 perfect synteny blocks identified in this study (as shown above, 51.1% genomic coverage). More recent studies have shown that operons are not as conserved as previously reported and that there is a greater turnover of operon composition among *Caenorhabditis *species [25,26], suggesting that the contribution of operons to the perfect synteny blocks between *C. elegans *and *C. briggsae *is even lower.

Second, if we define an operonic synteny block as a perfect synteny block with at least half of its genes being conserved operons, we find 385 such operonic synteny blocks (Figure 4). These operonic syntenic blocks contain 498 operons (or 44.5% of the total operons). These 385 operonic synteny blocks cover only 7.4% of the *C. elegans *genome, still much smaller than the 51.1% of the *C. elegans *genome covered by all perfect synteny blocks.

Third, the limited contribution of operons to the observed synteny is further illustrated by the low coverage of the X Chromosome by operons (2.1%, 57 operons) in *C. elegans*, which is the chromosome that is most covered by perfect synteny blocks (65.4%, 431 perfect synteny blocks) between *C. elegans *and *C. briggsae *(Table 4).

#### Imperfect synteny blocks

**Figure 5:**
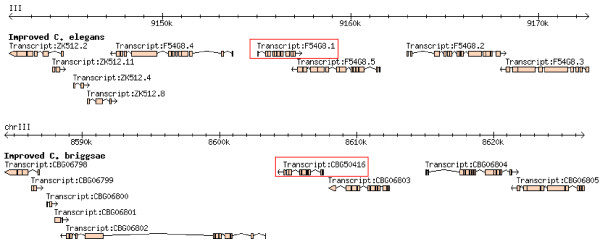
**Interruption of synteny block by disruption of strandedness**. This region in Chromosome III of the *C. elegans *genome contains nine genes, all of which have one-to-one relationships to their orthologous genes in a syntenic region in *C. briggsae*. The perfect synteny is disrupted by one gene, F54G8.1, whose ortholog is inverted in *C. briggsae*. The two adjacent perfect synteny blocks are merged into one large synteny block when we allow strandedness of genes to vary.

During evolution, genome sequences are often interrupted by small genome rearrangement events such as insertions, deletions, inversions and duplications. It has been suggested that small inversions and transpositions can be regarded as noise in genome rearrangements [27]. Identification of imperfect synteny blocks is valuable because they provide a global view of the existing synteny between different species for regions that have been subject to various types of rearrangement events. To detect such synteny blocks, we ran OrthoCluster by allowing mismatches (see methods) as well as by relaxing the constraints of order and strandedness of the genes within the blocks. In general, relaxing the constraints regarding gene order, strandedness and mismatches generates larger and fewer synteny blocks when compared to the perfect synteny blocks. In contrast to relaxing the number of mismatches, relaxing the constraints of order and strandedness within blocks alone has only a weak impact on block size distribution, suggesting that insertions/deletions and long-range transposition events are much more common than inversion and short-range transposition events. One example of a larger synteny block found when relaxing only order and strandedness constraints is one with 9 genes in Chromosome III of the *C. elegans *genome (Figure 5). This synteny block was split into two smaller ones when OrthoCluster was applied for detecting perfect synteny blocks. These two blocks, one of size 5 and the other 3, are separated by one in-map gene (F54G8.1) whose ortholog (CBG50416) is inverted with respect to the neighboring genes, hence disrupting the perfect conservation of strandedness.

**Figure 6:**
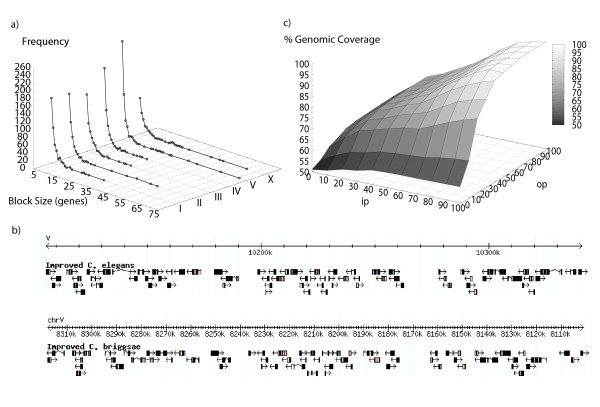
**Imperfect synteny blocks in *C. elegans***. a) Synteny blocks generated by allowing a maximal percentage of in-map and out-map mismatches of 5%. b) The largest imperfect synteny block between *C. elegans *(containing 71 genes) and *C. briggsae *(containing 68 genes). c) *C. elegans *genomic coverage of syntenic blocks as a function of both in-map and out-map mismatches.

**Figure 7:**
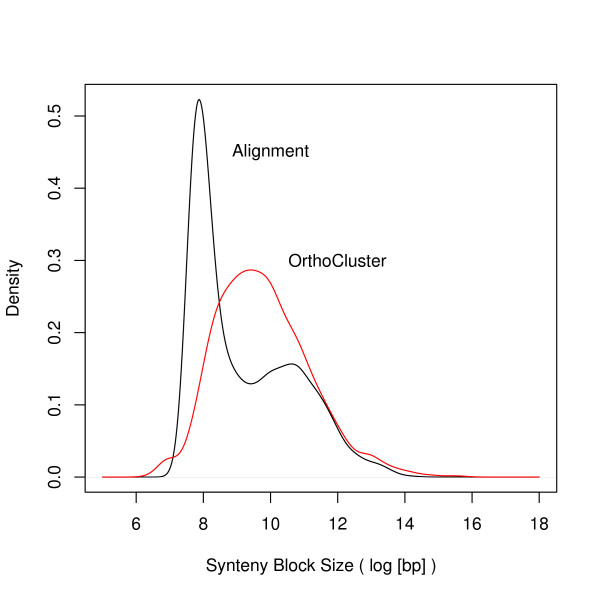
**Size distribution of synteny blocks between *C. elegans *and *C. briggsae***. The red curve represents synteny blocks identified using OrthoCluster (ip = 50%; op = 50%), while the black curve represents synteny blocks reported previously [19].

Allowing either in-map or out-map mismatches leads to the identification of larger synteny blocks because neighboring perfect synteny blocks start to merge. For example, using the improved annotation, when the percentage of both the in-map and the out-map mismatches are set to 5%, the largest block contains 71 genes (Figure 6a and 6b) (mean = 20.2 kb, median = 12.4 kb), compared to 42 genes identified as the largest block when no mismatches are allowed (Figure 1a; Figure 4). When these mismatch percentages are increased to 10% and 20%, the largest block contains 209 genes (mean = 26,7 kb, median = 12.0 kb) and 838 genes (mean = 45.1 kb, median = 14.1 kb), respectively. When we ran OrthoCluster by allowing a maximum of 50% in-map mismatch and 50% out-map mismatch within each synteny block, we found 80.8% of the genomic sequence of *C. elegans *being syntenic to 78.3% of the *C. briggsae *genomic sequence. As illustrated in Figure 6c, allowing more mismatches leads to merging of unrelated blocks because the genomic coverage increases sharply for mismatch percentages above this point. Also, for values larger than 50%, the number of synteny blocks decreases dramatically, mostly due the inclusion of in-map mismatches from unrelated regions of the genome (Additional file 7, Figure S4). At this setting, the median length of the synteny blocks found with this set of parameters is 15.6 kb (mean = 63.6 kb) (Figure 7). Again, the imperfect synteny blocks are not evenly distributed in the genomes. The mean size of imperfect synteny blocks is 53.6 kb (median = 15.7 kb) for autosomal synteny blocks, while 217.6 kb (median = 13.8 kb) for Chromosome X. This extremely large mean for the X chromosome compared to its median reflects that the size distribution of synteny blocks in the X chromosome is positively skewed (*i.e*., there are few very large synteny blocks). Within autosomes, again we don't observe a significant difference between centers and arms (p-value = 0.42, Mann-Whitney Test), with the median length of autosomal centers being 15.3 kb (mean = 62.1 kb), whereas the median length of autosomal arms is 16.6 kb (mean = 45.4 kb). This is in agreement with a previous report [5]. The largest synteny block spans 6.14 Mb on Chromosome X of *C. elegans*, between 1.68 Mb and 7.82 Mb. Altogether, there are 11 synteny blocks that are larger than 1 Mb between these two genomes. They are distributed across all chromosomes of *C. elegans *except Chromosome I and III. These 11 largest synteny blocks add up to 26 Mb. These large synteny blocks are unlikely to be found by chance under a random breakage model, even after correcting for multiple testing (data not shown) [28]. There are altogether 161 synteny blocks that are larger than 100 Kb, which add up to 66 Mb in size, strongly suggesting that *C. elegans *and *C. briggsae *genomes share large synteny blocks. As shown in Figure 7, synteny blocks identified here are significantly larger that those identified using an alignment-based approach [19].

## Discussion

In this work we applied our newly developed tool, OrthoCluster, for the detection of synteny blocks between the genome of *C. elegans *and the newly reconstructed *C. briggsae *genome. This anchor-based program has a number of features that makes it useful for identifying synteny blocks. In addition to identifying mismatches within syntenic regions, it takes into consideration one-to-many orthologous relationships at the moment of identifying synteny blocks. It is also sensitive to gene strandedness. More importantly, OrthoCluster works with multiple genomes so that users can explore synteny among the expanding number of sequenced genomes. Now that the genomes of three additional *Caenorhabditis *species (*C. remanei*, *C. japonica*, and *C. brenneri*) have been sequenced, we are eager to apply OrthoCluster to identify and analyze synteny relationships among these genomes. The appropriate handling of these types of features enables users to detect genome rearrangement events such as insertions, deletions, duplications, inversions, and reciprocal translocations. Furthermore, OrthoCluster can be used for the detection of segmental duplications within a single genome [15]. Since OrthoCluster is an anchor-based program, correct annotation of the genetic markers coordinates used as anchors is an essential condition for the accurate estimation of synteny. Taken together, OrthoCluster is a flexible tool for the detection of synteny blocks among species of different evolutionary distance.

We have demonstrated that syntenic information is useful for the improvement of defective gene models and detection of potential new genes and missing orthologous relationships. In this attempt, we have identified 582 new gene models (Table 1) in *C. briggsae *and 52 candidate new gene models in *C. elegans*. These improved annotations enabled us to identify 949 new orthologous relationships. Some of the new gene models that we have identified were independently detected by WormBase curators. For example, gene C10A4.10 was absent in WormBase release WS180, but was later curated and released in WS190. This gene was detected also with our procedure (Additional file 8, Figure S5).

The improved genome annotations and orthologous relationships have helped the synteny block analysis since larger synteny blocks are found in contrast to those obtained with WS180 annotations (Figure 1). Also, some conserved operon structures are restored with the improved annotations (Additional file 9, Figure S6). This methodology will be applied for improving the annotation of the newly sequenced genomes of *C. remanei*, *C. brenneri*, and *C. japonica*.

Hillier and colleagues constructed the first chromosomal level assembly of *C. briggsae *[21]. Taking advantage of OrthoCluster and this newly constructed *C. briggsae *assembly, we found that 80.8% of the *C. elegans *genome (and correspondingly 78.3% of the *C. briggsae *genome) is covered by synteny blocks that contain at least two genes. The amount of genome coverage by synteny blocks is consistent with a previous report [19]. Including "synteny blocks" composed of a single gene (in-map genes) only slightly increases the coverage of the *C. elegans *genome to 84.4% (corresponding to 81.9% of the *C. briggsae*). This coverage is also in excellent agreement with the work of Stein and colleagues (84.6% for *C. elegans *and 80.8% for *C. briggsae*) [19]. Thus, the conservation observed between the *C. elegans *and *C. briggsae *genomes is accounted for largely by synteny blocks that contain two or more genes. However, the synteny blocks discovered between *C. elegans *and *C. briggsae *using OrthoCluster (median size of 15.6 kb, average size of 63.6 kb) are much larger than those identified by the previous whole genome analysis (median size of 5.6 kb, average size of 37.5 kb).

## Conclusions

Taken together, we have demonstrated that OrthoCluster can be used to accurately identify synteny blocks. Additionally, we have found that synteny blocks between *C. elegans *and *C. briggsae *are almost three-folds larger than previously identified.

## Methods

### OrthoCluster

OrthoCluster algorithm and development was described previously [6]. Briefly, it uses an anchor-based approach to effectively search for synteny blocks between two or more genomes given parameters for controlling synteny block size, mismatches within synteny blocks as well as preservation of order and strandedness (Additional file 10, Figure S7). Since OrthoCluster takes into consideration both order and strandedness of genes, it is useful for the detection of inversions and other genome rearrangement events. In addition to identifying perfect synteny blocks (that contain no mismatches and preserve gene order and strandedness), it can be applied to identify imperfect synteny blocks with various levels of mismatches. OrthoCluster needs two types of input files (Additional file 11, Figure S8): a genome file and a correspondence file. A genome file contains genetic markers (which could be annotated genes) with information regarding chromosome/supercontig names, start and end positions, as well as the strand in which each genetic marker resides. A correspondence file provides orthologous relationships between two (for pair-wise analysis) or more genomes (for multiple-genomes analysis). Genetic markers that are not included in the correspondence file are called out-map genetic markers (in this paper, "genes" and "genetic markers" are used interchangeably). In contrast, genetic markers that are part of the correspondence file are called in-map genetic markers. A synteny block can be non-nested or nested (Additional file 12, Figure S9) with nested block defined as one that is contained within a larger block. A nested synteny results from a segmental duplication of a portion of a larger synteny block in one genome (Additional file 12, Figure S9d).

### Data Sources

Genome annotations of *C. elegans *and *C. briggsae *were obtained from WormBase http://www.wormbase.org/[17], release WS180. Since some genes produces multiple alternative isoforms and all of these isoforms represent one gene (locus), we used the longest isoform to represent a gene.

#### Correspondence file preparation

To generate the correspondence file required by OrthoCluster, we assigned orthologous relationships between different genomes using InParanoid [22,29] with default settings. InParanoid has been evaluated to be one of the best performing methods for orthology detection [29]. Ortholog assignment between *C. elegans *and *C. briggsae *is further improved based on gene model improvement, sequence similarity, and synteny when applying our gene model improvement procedure. A correspondence file contains both one-to-one and one-to-many relationships.

#### Synteny based gene model improvement and ortholog assignment

**Figure 8:**
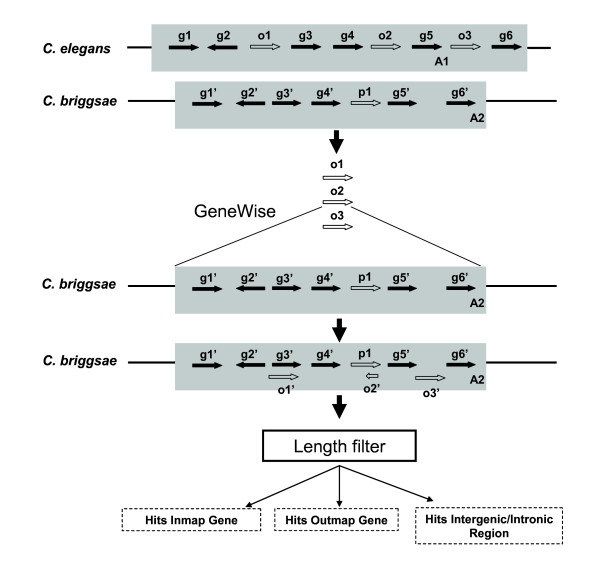
**Synteny-based gene model improvement procedure**. First, out-map mismatches are identified in the synteny blocks. Second, GeneWise is run to identify candidate genes using out-map mismatches as queries and the corresponding syntenic region as target. Third, predicted genes are examined and compared with other genes in the synteny blocks (proteins encoded by the predicted genes are at least 60% as long as their corresponding query proteins).

As illustrated in Figure 8, we first identified imperfect synteny blocks that contain out-map mismatch genes using OrthoCluster. Out-map mismatches, which usually indicate genome-specific genes, can also indicate these two alternative possibilities: (1) the ortholog gene in the other genome has not been found, and (2) the corresponding gene model is defective in a way the orthologous relationship can't be established by orthology detection programs. Synteny information helps narrow down genomic regions that contain these missing or defective orthologous genes and improve defective gene models. Once we identified mismatches in synteny blocks, we attempted to identify missing/defective gene models using the homology-based gene prediction method GeneWise [30,31]. When we ran OrthoCluster by allowing up to 20 out-map mismatches per synteny block, we found 2,650 imperfect synteny blocks, 2,389 of which are non-nested blocks and 261 are nested ones. Of the 1,886 out-map mismatch genes within synteny blocks in the *C. elegans *genome, 695 *C. elegans *genes generated GeneWise predictions in *C. briggsae *that satisfy the filtration criteria described below (Additional file 13). We only consider predictions that cover at least 60% of the length of the query proteins with no internal stop codons. We identified 771 GeneWise predictions in *C. briggsae *genome. Note that some out-map mismatch genes generate more than one valid prediction (paralogs) within the corresponding synteny block. Applying the same strategy, we identified 702 GeneWise predictions in *C. elegans*. Depending on which location of the synteny block the prediction hits, each of the predictions can be categorized accordingly. There are two possibilities. First, the predicted gene overlaps with an intergenic or intronic region. In this case, we take the predicted gene as a new candidate gene. Second, the predicted gene overlaps with one or more existing genes within the corresponding synteny block (Additional file 14, Figure S10).

We also assigned new orthologous relationships using synteny information and similarity (blast alignment scores). To achieve this, we compared the out-map genes with the new gene models and calculate their percentage identity (PID). We accept a new pair of orthologs if the PID between them is greater than or equal to 40% and the e-value is less or equal than 1e-10. The revised orthologous relationships were then incorporated into the InParanoid-driven orthologous relationships.

## Authors' contributions

NC conceived of the study. IAV conducted the experiments and NC and IAV wrote the manuscript. All authors have read and approved the final manuscript.

## Supplementary Material

Additional file 1 **new gene models for *C. elegans*. **gff3 file with the structure of all new genes in *C. elegans*. Click here for file

Additional file 2 **new genome annotation for *C. briggsae***. gff3 file with the structure of all genes in the new genome annotation for *C. briggsae *New genes start with ID CBG5XXXX. Click here for file

Additional file 3 **Figure S1 genome view of the perfect synteny blocks between *C. elegans *and *C. briggsae***. Each chromosome in *C. elegans *has a distinctive color. The corresponding synteny blocks in *C. briggsae *can be mapped to the reference chromosome according to the color. This image was created using OrthoClusterDB http://genome.sfu.ca/orthoclusterdb/. Click here for file

Additional file 4 **Figure S2 an example of syntenic tandem gene expansion/contraction**. A GST tandem gene cluster in *C. elegans *has nine genes, while its orthologous region in *C. briggsae *has four genes. Click here for file

Additional file 5 **Figure S3 Cumulative distribution of perfect synteny blocks in *C. elegans***. Black bars represent perfect synteny blocks found using WS180 annotation, while empty bars represent perfect synteny blocks found using improved annotation. Click here for file

Additional file 6 **Perfect synteny blocks and their corresponding genomic coverage in *C. elegans *for the improved and the WS180 annotations**. Click here for file

Additional file 7 **Figure S4 *C. elegans *distribution of the number of syntenic blocks as a function of both in-map and out-map mismatches**. Click here for file

Additional file 8 **Figure S5 A new gene model in *C. elegans***. This new gene model, absent in WS180, was reported independently by WormBase curators in WS190 and found with our methodology. Click here for file

Additional file 9 **Figure S6 Conserved operon revealed by improved genome annotation**. The improved annotation of C. briggsae identified two putative genes, CBG50308 and CBG50462, which are orthologs to the operonic genes C14A4.1 and C14A4.4, that were missing orthologs previous to the application of the gene model improvement procedure. Click here for file

Additional file 10 **Figure S7 Different types of order and strandedness handled by OrthoCluster**. a) Consistent order and consistent strandedness. Blocks A1 in genome G1 and A2 in genome G2 are composed of four genes. The order of the genes within each block is the same, and each pair of genes has the same orientation. b) Consistent order, reversed strandedness. Blocks A1 in genome G1 and A2 in genome G2 are composed of four genes. The order of the genes within each block is the same, but each pair of genes has different orientation. c) Inverted order, consistent strandedness. Blocks A1 in genome G1 and A2 in genome G2 are composed of four genes. The order of the genes within block A1 is inverted with respect to that within block A2, and each pair of genes has the same orientation. d) Inverted order, reversed strandedness. Blocks A1 in genome G1 and A2 in genome G2 are composed of four genes. The order of the genes in block A1 is inverted with respect to that in block A2, and each pair of genes has different orientation. All four cases are found if the user sets -r -s when running OrthoCluster. Cases a) and d) are found only if user sets -rs when running OrthoCluster. For the synteny blocks detected in this work, the parameter -rs was used. Click here for file

Additional file 11 **Figure S8 input and output data for OrthoCluster**. The input of the program consists of the genome annotation for each species (gene name, Chromosome/Contig, Start position, End Position, and Strand) and a correspondence file with the orthologous relationships among genes. The output corresponds to the synteny blocks found. In ths example, there are N genomes and a region of M genes is shown for each one. Click here for file

Additional file 12 **Figure S9 out-map and in-map mismatches**. a) An out-map mismatch. Given the corresponding syntenic regions A1 and A2 in genomes G1 and G2 respectively, A1 contains a gene (shown in white) that has no correspondence in G2. b) An in-map mismatch. Given the corresponding syntenic regions A1 and A2 in genomes G1 and G2 respectively, A1 contains a gene, g5, which has a correspondence in G2, but is distant enough from the other genes conforming A2 so it can not be include within the synteny block. Different numbers of in-map and out-map mismatches can be included in each block by varying the parameters -i, -ip, for in-map mismatches, and -o, -op for out-map mismatches. c) A non-nested synteny block. Blocks A1 and B1 in genome G1 are located in different regions of the genome, and the corresponding regions A2 and B2 in genome G2 are also located in different regions. d) A nested synteny block. Block B1 in genome G1 is fully contained within block A1, but the corresponding syntenic regions B2 and A2 in genome G2 are located in different regions of that genome. Click here for file

Additional file 13 **out-map mismatches used for gene model improvement**. Numbers in parentheses represent the number of unique genes that are associated to each number of mismatches. Click here for file

Additional file 14 **Figure S10 Gene model improvement procedure for the reparation of genes**. If the prediction hits a gene, then different procedures are defined depending on the gene been an in-map or out-map gene. If the gene hit is an in-map gene, then we measure the genomic coverage of the in-map gene. If the coverage is greater or equal than the threshold defined, then the prediction is discarded. If the coverage is less than the threshold, then the peptide of the ortholog of g1', g1, is used as query against the genomic span if g1'. If the predictions overlap, then they are discarded. If the predictions do not overlap and g1' is in *C. briggsae*, then g1' is replaced by o1' and g1''. If g1' is in *C. elegans*, then its peptide is used as query against the genomic span of g1 - o1 in *C. briggsae *to determine if those genes can be merged (g1'''). If the prediction hits an out-map gene and the coverage is less than the original gene model, then the prediction is discarded. If the coverage is greater or equal than the original gene model, then o1' is discarded if p1 is located in *C. elegans*. If p1 is located in *C. briggsae*, the prediction o1' replaces p1. Click here for file
