# Supplementary figures and images for: Large synteny blocks revealed between Caenorhabditis elegans and Caenorhabditis briggsae genomes using OrthoCluster
Source: BMC Genomics. 2010 Sep 24;11:516. doi: 10.1186/1471-2164-11-516 (PMC2997010; doi:10.1186/1471-2164-11-516)

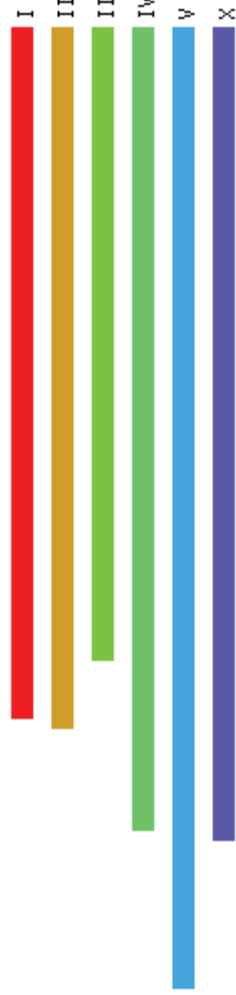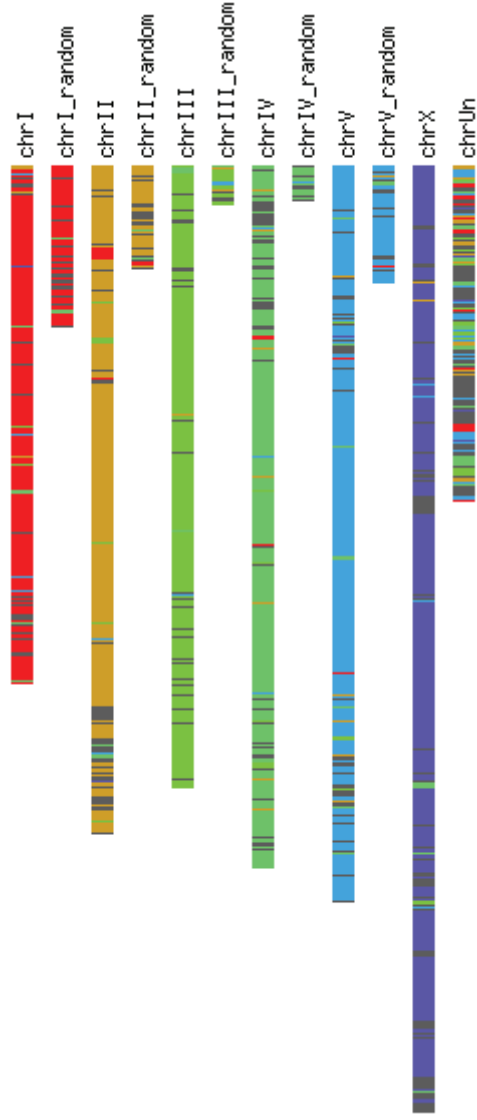

Supplement: Additional file 3 — Figure S1 genome view of the perfect synteny blocks between C. elegans and C. briggsae. Each chromosome in C. elegans has a distinctive color. The corresponding synteny blocks in C. briggsae can be mapped to the reference chromosome according to the color. This image was created using OrthoClusterDB http://genome.sfu.ca/orthoclusterdb/. [file 1471-2164-11-516-S3.PDF]

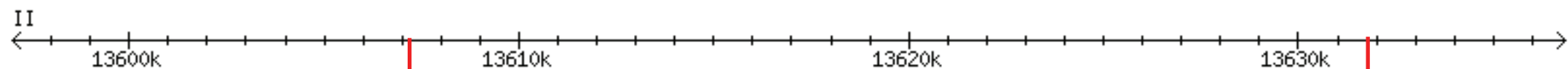

### Gene Models

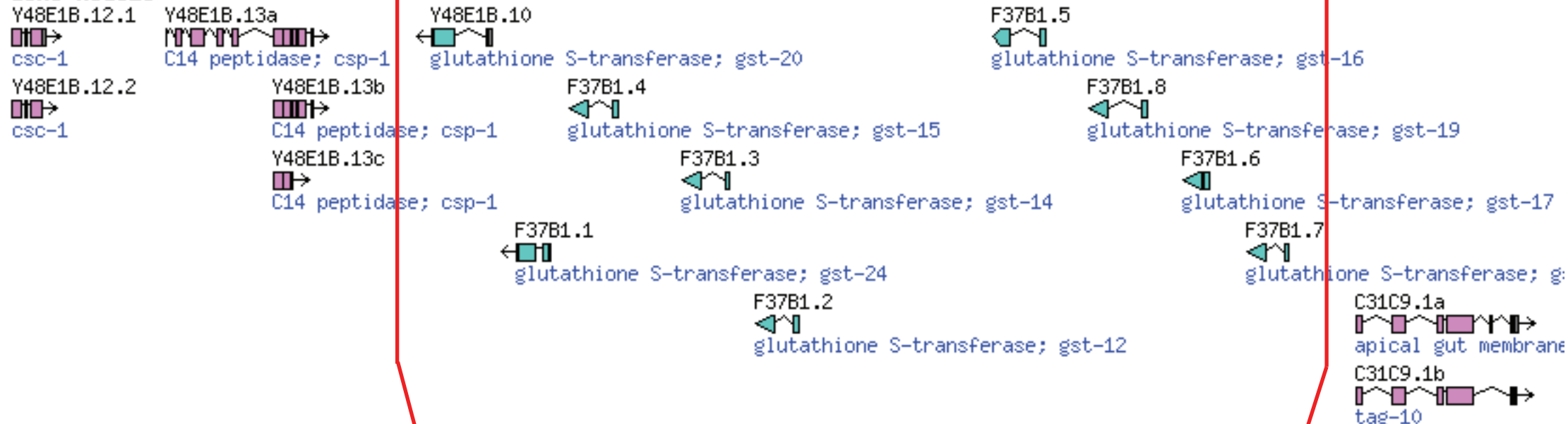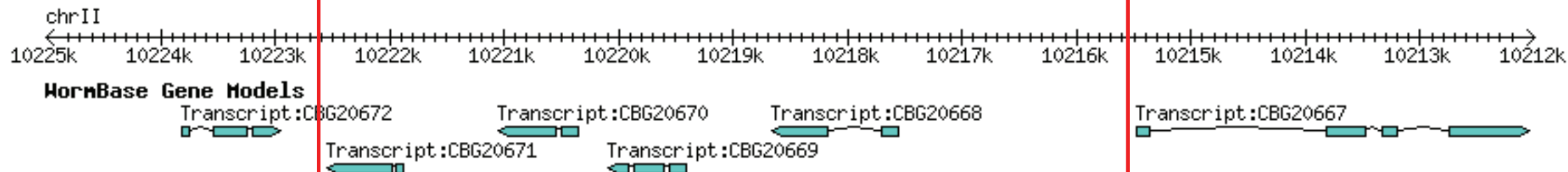

Supplement: Additional file 4 — Figure S2 an example of syntenic tandem gene expansion/contraction. A GST tandem gene cluster in C. elegans has nine genes, while its orthologous region in C. briggsae has four genes. [file 1471-2164-11-516-S4.PDF]

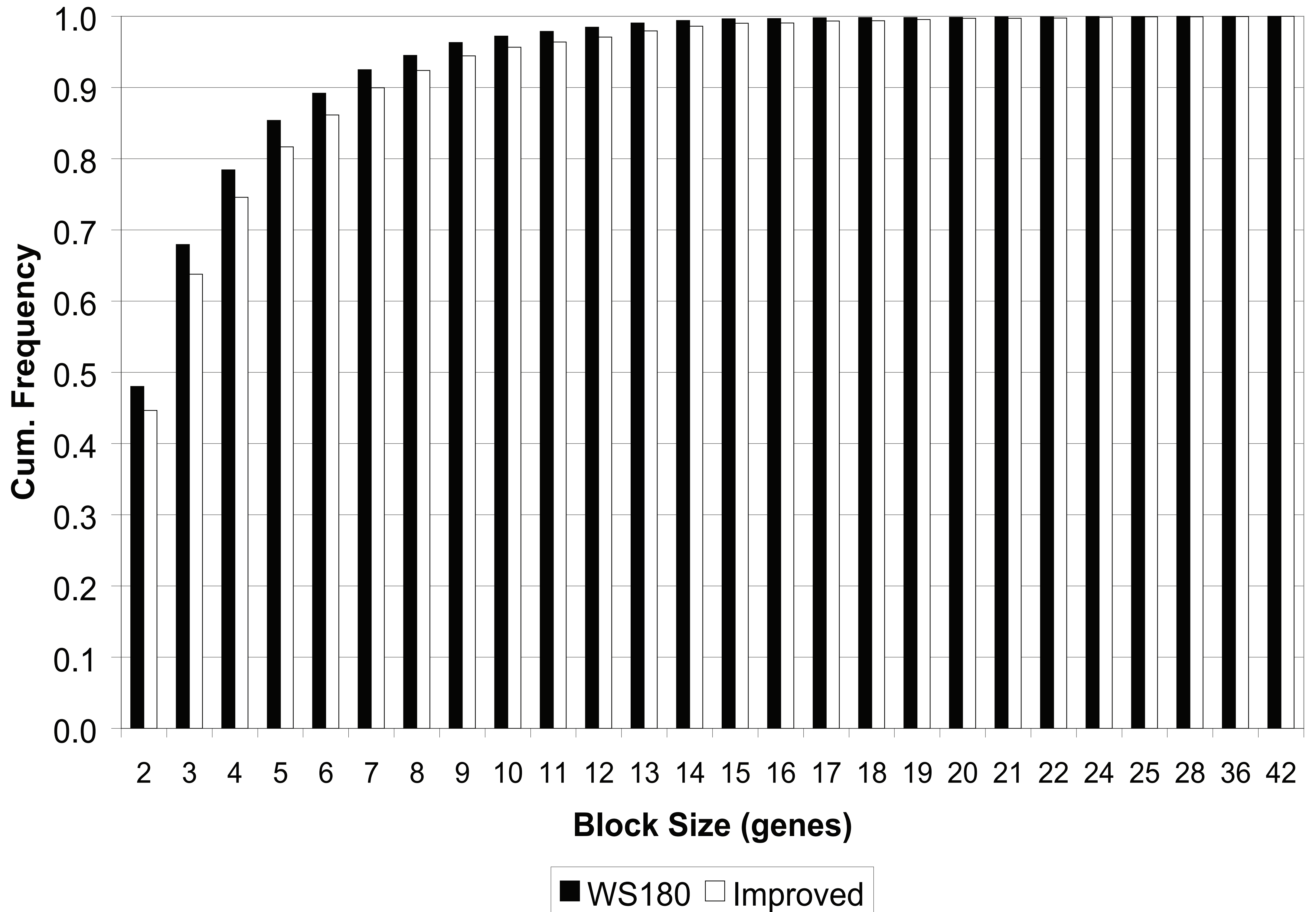

Supplement: Additional file 5 — Figure S3 Cumulative distribution of perfect synteny blocks in C. elegans. Black bars represent perfect synteny blocks found using WS180 annotation, while empty bars represent perfect synteny blocks found using improved annotation. [file 1471-2164-11-516-S5.PDF]

# Number Synteny Blocks

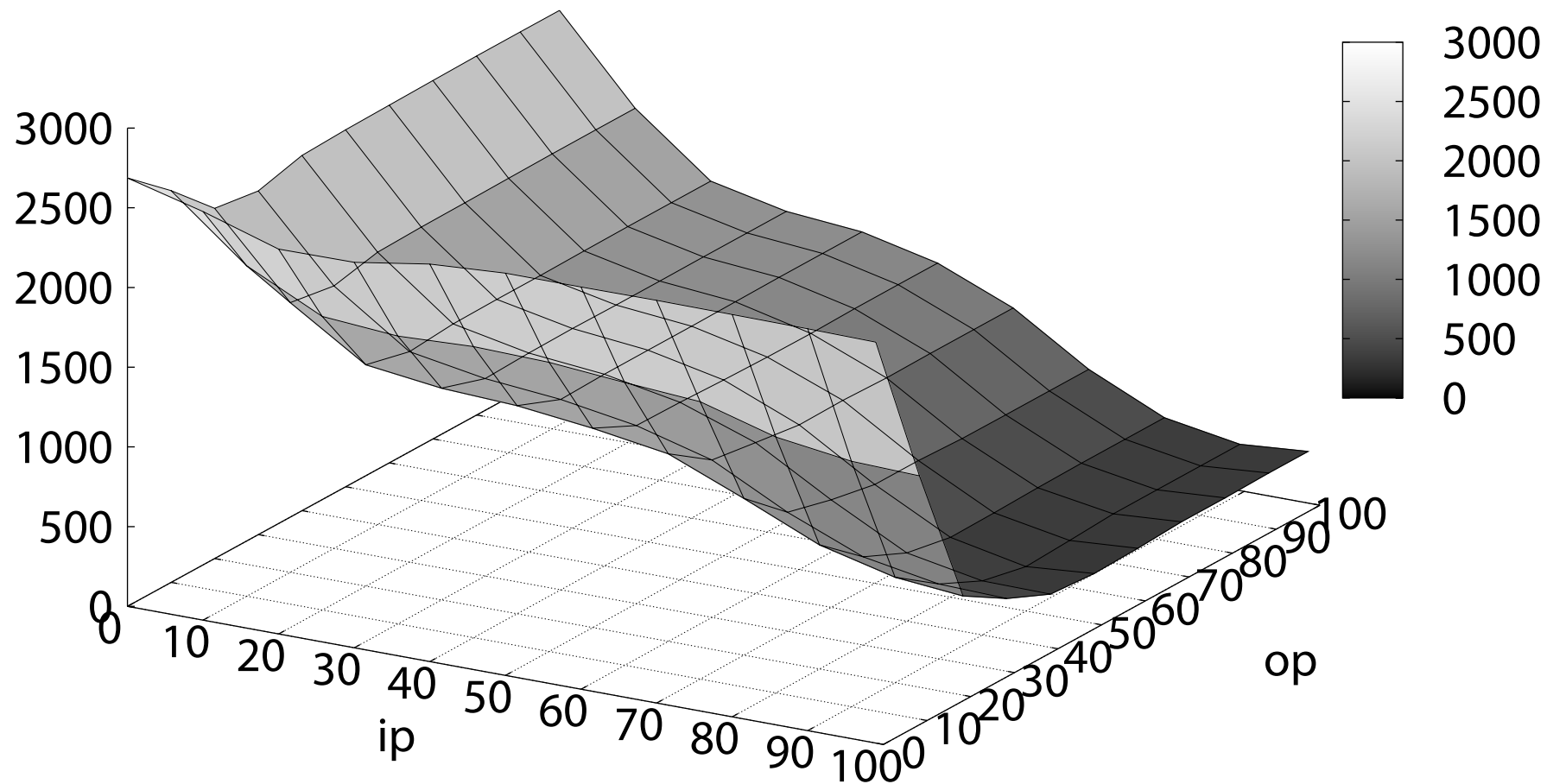

Supplement: Additional file 7 — Figure S4 C. elegans distribution of the number of syntenic blocks as a function of both in-map and out-map mismatches. [file 1471-2164-11-516-S7.PDF]

## *C. elegans*

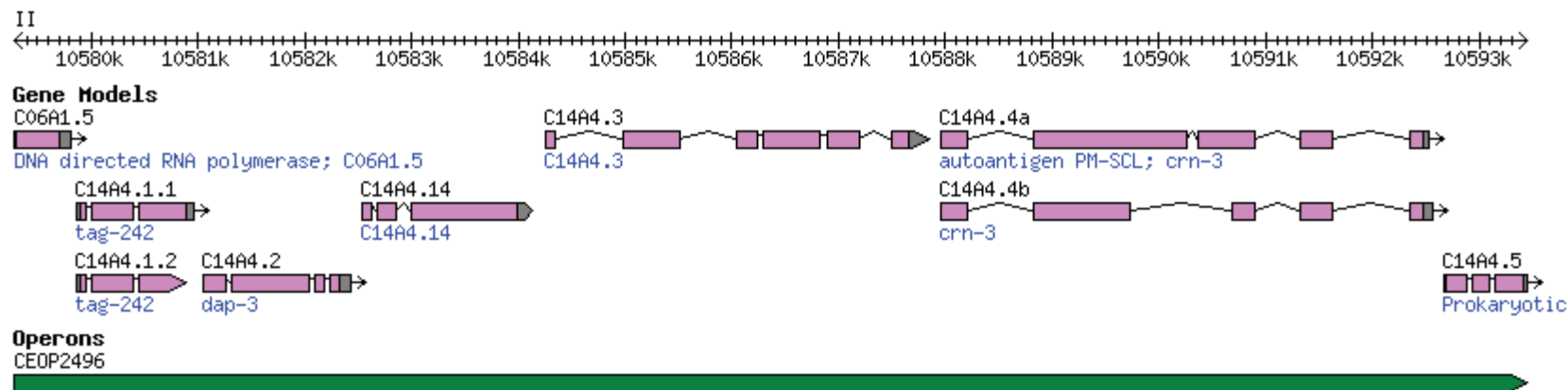

## *C. briggsae*

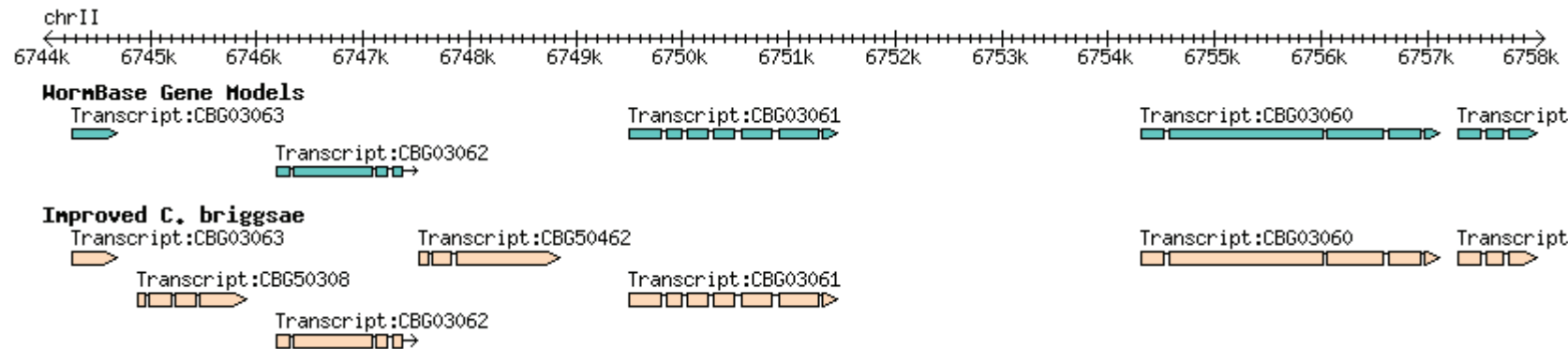

Supplement: Additional file 9 — Figure S6 Conserved operon revealed by improved genome annotation. The improved annotation of C. briggsae identified two putative genes, CBG50308 and CBG50462, which are orthologs to the operonic genes C14A4.1 and C14A4.4, that were missing orthologs previous to the application of the gene model improvement procedure. [file 1471-2164-11-516-S9.PDF]

a)

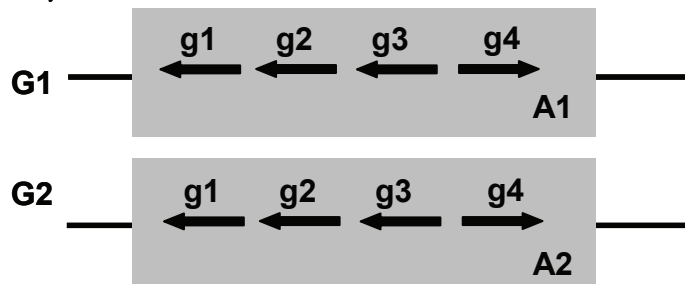

b)

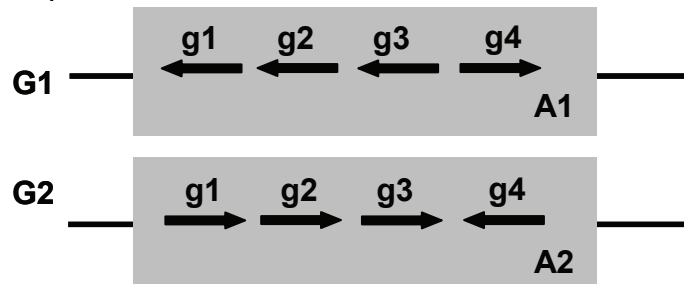

c)

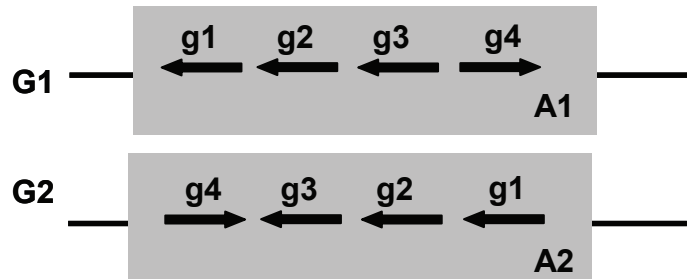

d)

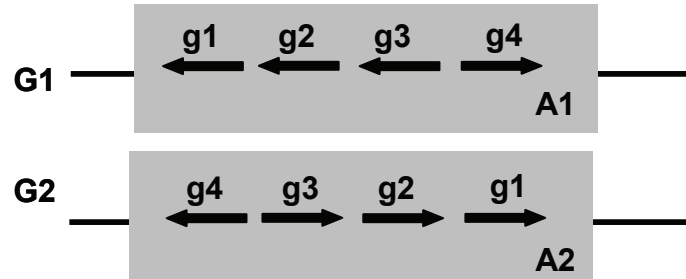

Supplement: Additional file 10 — Figure S7 Different types of order and strandedness handled by OrthoCluster. a) Consistent order and consistent strandedness. Blocks A1 in genome G1 and A2 in genome G2 are composed of four genes. The order of the genes within each block is the same, and each pair of genes has the same orientation. b) Consistent order, reversed strandedness. Blocks A1 in genome G1 and A2 in genome G2 are composed of four genes. The order of the genes within each block is the same, but each pair of genes has different orientation. c) Inverted order, consistent strandedness. Blocks A1 in genome G1 and A2 in genome G2 are composed of four genes. The order of the genes within block A1 is inverted with respect to that within block A2, and each pair of genes has the same orientation. d) Inverted order, reversed strandedness. Blocks A1 in genome G1 and A2 in genome G2 are composed of four genes. The order of the genes in block A1 is inverted with respect to that in block A2, and each pair of genes has different orientation. All four cases are found if the user sets -r -s when running OrthoCluster. Cases a) and d) are found only if user sets -rs when running OrthoCluster. For the synteny blocks detected in this work, the parameter -rs was used. [file 1471-2164-11-516-S10.PDF]

a)

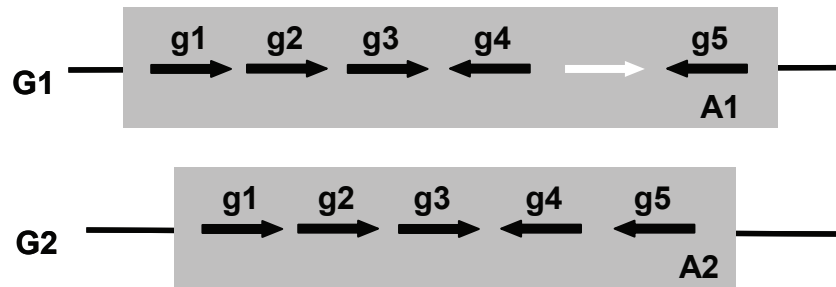

b)

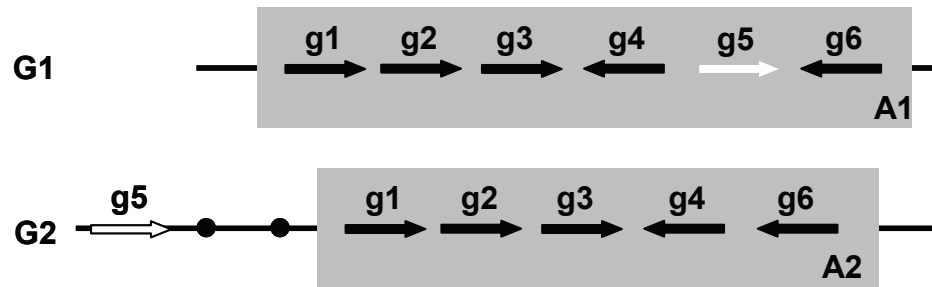

c)

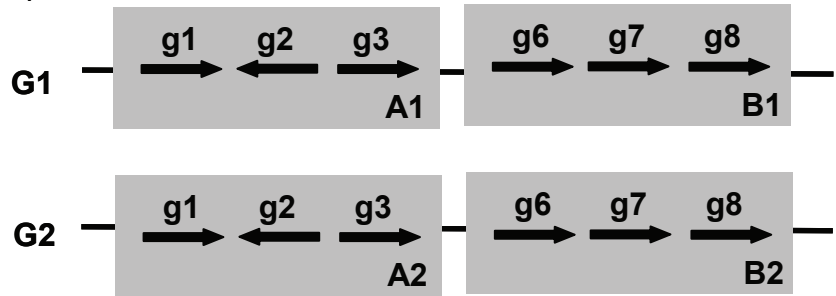

d)

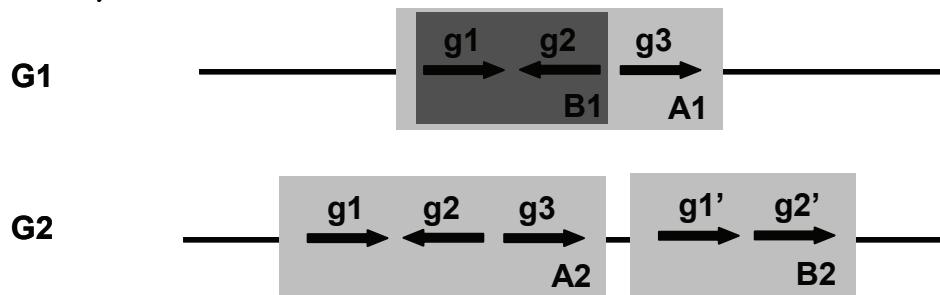

Supplement: Additional file 12 — Figure S9 out-map and in-map mismatches. a) An out-map mismatch. Given the corresponding syntenic regions A1 and A2 in genomes G1 and G2 respectively, A1 contains a gene (shown in white) that has no correspondence in G2. b) An in-map mismatch. Given the corresponding syntenic regions A1 and A2 in genomes G1 and G2 respectively, A1 contains a gene, g5, which has a correspondence in G2, but is distant enough from the other genes conforming A2 so it can not be include within the synteny block. Different numbers of in-map and out-map mismatches can be included in each block by varying the parameters -i, -ip, for in-map mismatches, and -o, -op for out-map mismatches. c) A non-nested synteny block. Blocks A1 and B1 in genome G1 are located in different regions of the genome, and the corresponding regions A2 and B2 in genome G2 are also located in different regions. d) A nested synteny block. Block B1 in genome G1 is fully contained within block A1, but the corresponding syntenic regions B2 and A2 in genome G2 are located in different regions of that genome. [file 1471-2164-11-516-S12.PDF]

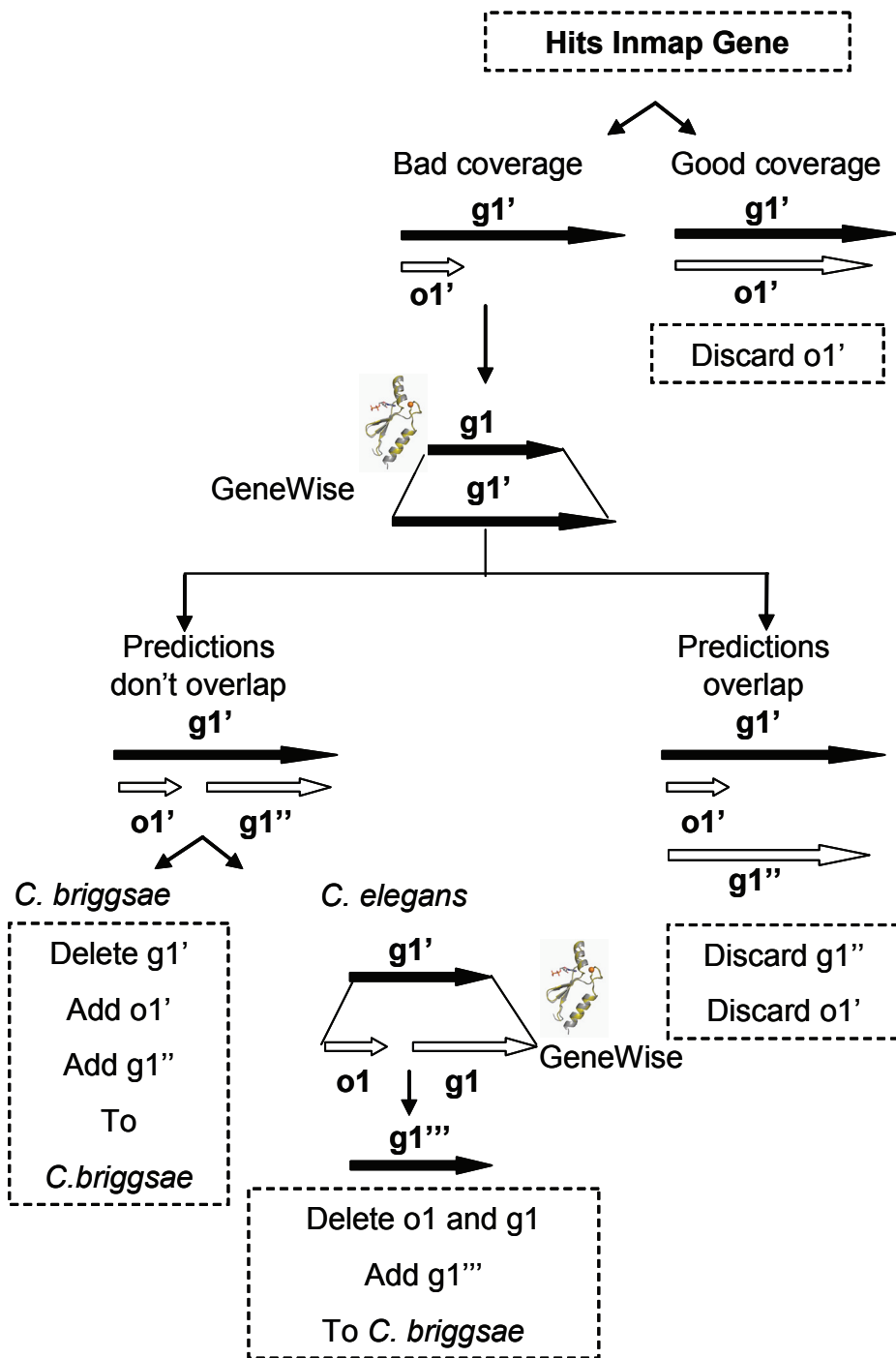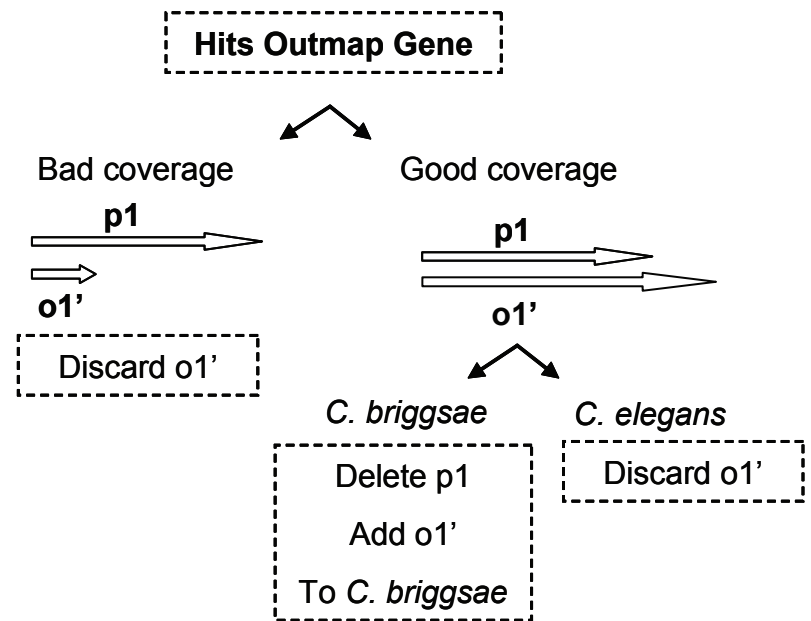

Supplement: Additional file 14 — Figure S10 Gene model improvement procedure for the reparation of genes. If the prediction hits a gene, then different procedures are defined depending on the gene been an in-map or out-map gene. If the gene hit is an in-map gene, then we measure the genomic coverage of the in-map gene. If the coverage is greater or equal than the threshold defined, then the prediction is discarded. If the coverage is less than the threshold, then the peptide of the ortholog of g1', g1, is used as query against the genomic span if g1'. If the predictions overlap, then they are discarded. If the predictions do not overlap and g1' is in C. briggsae, then g1' is replaced by o1' and g1''. If g1' is in C. elegans, then its peptide is used as query against the genomic span of g1 - o1 in C. briggsae to determine if those genes can be merged (g1'''). If the prediction hits an out-map gene and the coverage is less than the original gene model, then the prediction is discarded. If the coverage is greater or equal than the original gene model, then o1' is discarded if p1 is located in C. elegans. If p1 is located in C. briggsae, the prediction o1' replaces p1. [file 1471-2164-11-516-S14.PDF]
