# Supplementary material for: Large synteny blocks revealed between Caenorhabditis elegans and Caenorhabditis briggsae genomes using OrthoCluster
Source: BMC Genomics. 2010 Sep 24;11:516. doi: 10.1186/1471-2164-11-516 (PMC2997010; doi:10.1186/1471-2164-11-516)
Supplement: Additional file 6 — Perfect synteny blocks and their corresponding genomic coverage in C. elegans for the improved and the WS180 annotations. [file 1471-2164-11-516-S6.DOC]

Perfect synteny blocks and their corresponding genomic coverage in *C. elegans* for the improved and the WS180 annotations.
