# Supplementary material for: Large synteny blocks revealed between Caenorhabditis elegans and Caenorhabditis briggsae genomes using OrthoCluster
Source: BMC Genomics. 2010 Sep 24;11:516. doi: 10.1186/1471-2164-11-516 (PMC2997010; doi:10.1186/1471-2164-11-516)
Supplement: Additional file 8 — Figure S5 A new gene model in C. elegans. This new gene model, absent in WS180, was reported independently by WormBase curators in WS190 and found with our methodology. [file 1471-2164-11-516-S8.PDF]

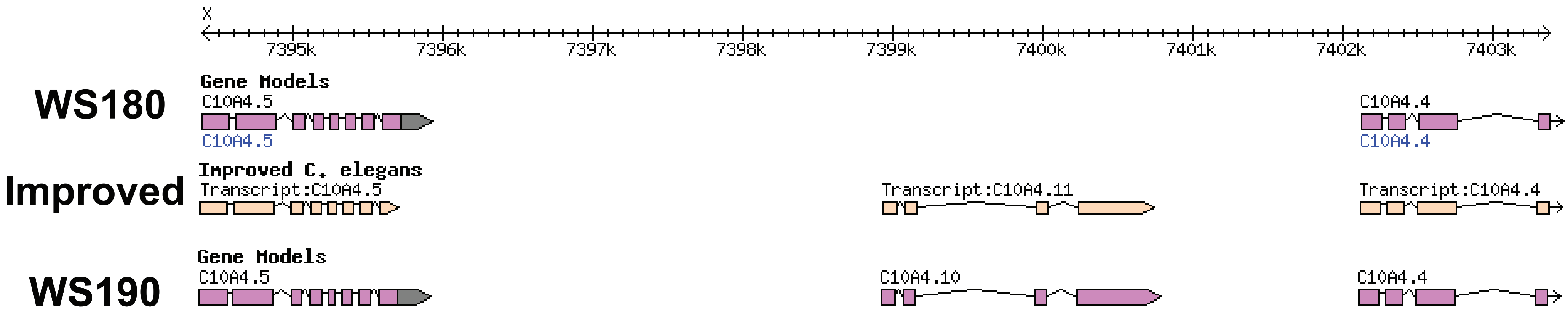

X
7395k
7396k
7397k
7398k
7399k
7400k
7401k
7402k
7403k
WS180
Gene Models
C10A4.5
C10A4.5
C10A4.4
C10A4.4
Improved
Improved C. elegans
Transcript:C10A4.5
Transcript:C10A4.11
Transcript:C10A4.4
WS190
Gene Models
C10A4.5
C10A4.10
C10A4.4
