# Supplementary material for: Large synteny blocks revealed between Caenorhabditis elegans and Caenorhabditis briggsae genomes using OrthoCluster
Source: BMC Genomics. 2010 Sep 24;11:516. doi: 10.1186/1471-2164-11-516 (PMC2997010; doi:10.1186/1471-2164-11-516)
Supplement: Additional file 11 — Figure S8 input and output data for OrthoCluster. The input of the program consists of the genome annotation for each species (gene name, Chromosome/Contig, Start position, End Position, and Strand) and a correspondence file with the orthologous relationships among genes. The output corresponds to the synteny blocks found. In ths example, there are N genomes and a region of M genes is shown for each one. [file 1471-2164-11-516-S11.PDF]

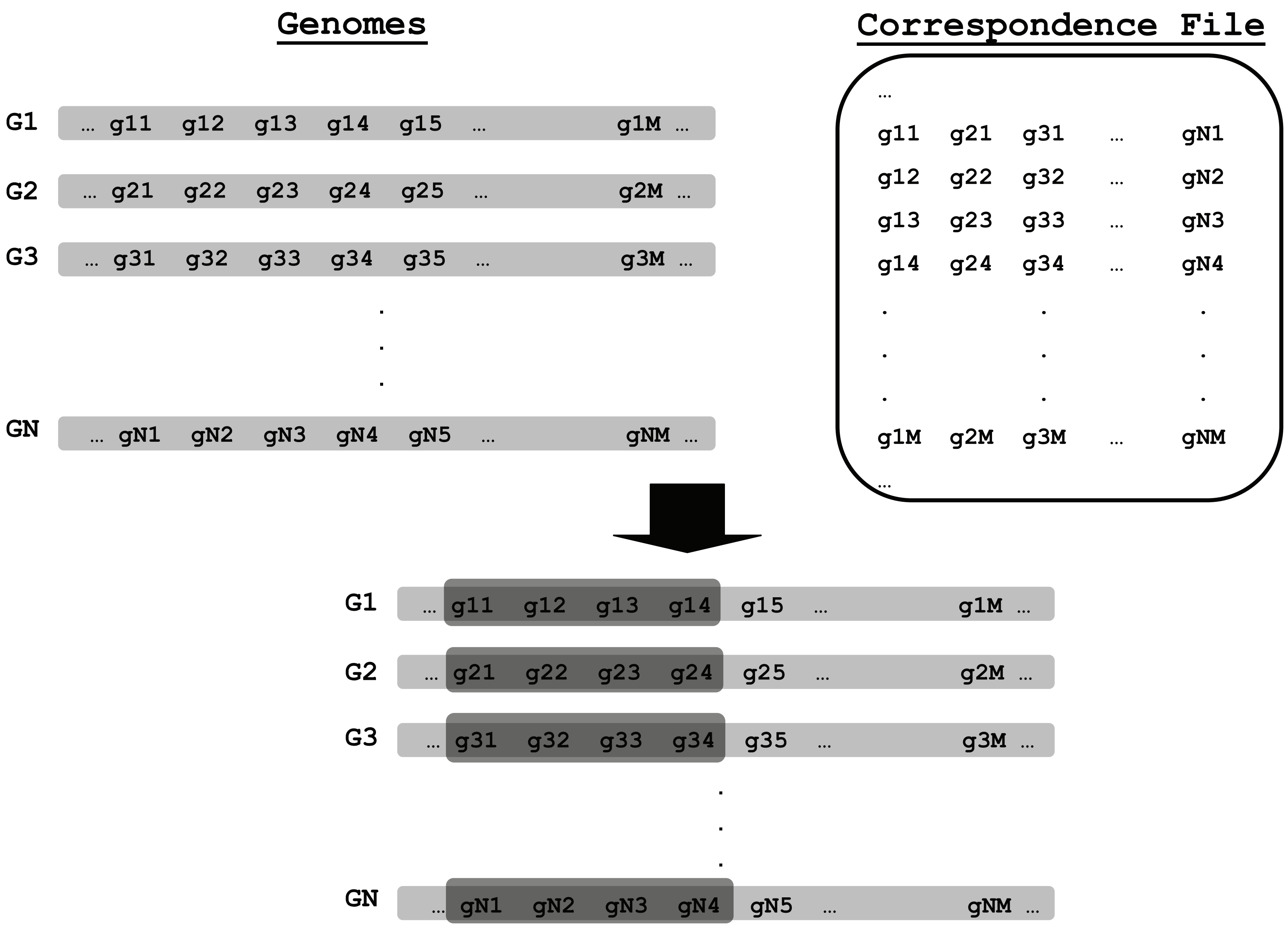

Genomes
Correspondence File
G1 … g11 g12 g13 g14 g15 … g1M …
G2 … g21 g22 g23 g24 g25 … g2M …
G3 … g31 g32 g33 g34 g35 … g3M …
GN … gN1 gN2 gN3 gN4 gN5 … gNM …
…
g11 g21 g31 … gN1
g12 g22 g32 … gN2
g13 g23 g33 … gN3
g14 g24 g34 … gN4
g1M g2M g3M … gNM
…
G1 … g11 g12 g13 g14 g15 … g1M …
G2 … g21 g22 g23 g24 g25 … g2M …
G3 … g31 g32 g33 g34 g35 … g3M …
GN … gN1 gN2 gN3 gN4 gN5 … gNM …
