# Supplementary material for: Large synteny blocks revealed between Caenorhabditis elegans and Caenorhabditis briggsae genomes using OrthoCluster
Source: BMC Genomics. 2010 Sep 24;11:516. doi: 10.1186/1471-2164-11-516 (PMC2997010; doi:10.1186/1471-2164-11-516)
Supplement: Additional file 13 — out-map mismatches used for gene model improvement. Numbers in parentheses represent the number of unique genes that are associated to each number of mismatches. [file 1471-2164-11-516-S13.DOC]

Out-map mismatches used for gene model improvement. Numbers in parenthesis represent the number of unique genes that are associated to each number of mismatches.
